# LED Railway Signal Detection Rather Than Recognition is Affected by Both Refractive and Non-refractive Blur

**DOI:** 10.1007/s44402-026-00035-1

**Published:** 2026-03-02

**Authors:** Jack Choy, Timothy Nguyen, Mei Ying Boon, Vanessa J. Honson, Stephen J. Dain

**Affiliations:** 1 School of Optometry and Vision Science, University of New South Wales, Sydney, New South Wales Australia; 2 Optometry and Vision Science, University of Canberra, Canberra, Australian Capital Territory Australia

**Keywords:** Blur, Colour vision, Occupational safety, Railway signals, Vision standards

## Abstract

**Purpose:**

Previous research has reported a significant effect of refractive defocus on the correct identification of red signals. The purpose of this study is to investigate the effects of both refractive defocus and non-refractive defocus (using Bangerter filters) on the perception of rail signals using the Railway LED Lantern Test (RLLT). The RLLT is the simulated practical test nominated in the Australian National Standard for Health Assessment of Railway Safety Workers.

**Method:**

Participants were 19–59 years old, and the best corrected visual acuity (BVCA) was required to be no worse than 6/9 binocularly. Subjects with current or active ocular conditions were excluded, and sufficiency in English was required. Best corrected refraction, visual acuity and colour vision were assessed. Participants carried out the RLLT binocularly under five conditions: best corrected, +0.50 DS, +0.75 DS and Bangerter filters 1.0 and 0.8.

**Results:**

Ten male and 10 female subjects completed the study; age range 20–25 and mean age 22.4 ± 1.1 years. BVCA was 6/6 (logMAR 0.0 or better).

Errors occurred far more often with red than with yellow or green (*p* < 0.0001) and with Bangerter filter blur more than refractive blur (*p* < 0.0001). Failing to see a red signal, rather than misnaming the red as yellow or green, was the predominant error (*p* < 0.0001) and was induced far more frequently by Bangerter filters than refractive blur (*p* < 0.0001). This error was far more common than miscalling red as yellow (*p* < 0.0001) (paired *t*-tests).

**Conclusion:**

These findings suggest that a large proportion of errors are due to not seeing the red signal rather than miscalling the red as yellow or green. Non-refractive blur was found to cause a greater increase in colour errors.

Key Points

- Signal colour detection is impeded by visual acuity reduction more effectively when the blur is created with Bangerter filters compared with plus-powered lenses, but the mechanism is not known.
- The detection and recognition of red signals are significantly more affected than green signals, and yellow signals are the least affected.
- It is not yet possible to relate these observations to the signals passed at danger events that occur on railways because the necessary questions are not routinely asked in the enquiry after the event.

## Introduction

Signals passed at danger (SPAD) are events where a train passes a stop signal without authority [1, 2]. Previous research has reported refractive defocus as being a significant factor in signal recognition that can occur when looking through low added power lenses, such as the corridor of progressive addition lenses (PALs), when the signal lights are viewed against a bright background or the sun is a significant glare source [3, 4].

A survey of Australian and New Zealand rail organisations found that SPADs occur up to 62 times annually, resulting in associated delays averaging 270 min costing $AU153,000 and are accompanied by preventative and reactive costs of up to $AU520,060 and $AU1.87 million annually, respectively [5]. A previous study in the 1980s reported no significant situational or personal factors underlying 224 SPAD events in the Netherlands [6]. Weather and adverse conditions such as darkness, rain and fog did not contribute to SPADs. These events were, however, more frequent in the morning, particularly 4–9 am, and at the start of locomotive drivers’ duty periods. The authors alluded to fatigue as possibly being the major factor. Drivers committing SPADs also had lower reaction scores on a multi-choice reaction test and less occupational satisfaction.

The effects of optical defocus on rail signals misperception were studied after a report by a train driver that red signals appeared yellow when viewed at long distances through PALs but not his bifocals [3]. They reported that they assessed him as having normal colour vision, but the tests used did not include an anomaloscopy, so the possibility of his being a very mild deuteranomal remains. Studies by Wood et al. [3, 4] found a significant percentage of red signals reported as appearing orange-yellow (they did not use ‘yellow’ as the alternative to ‘red’) in the presence of a bright background or glare source, at longer distances and through spherical refractive blur in both field and laboratory environments. The follow-up study [4] involved a change in protocol testing using signals satisfying the Australian Rail Track Corporation Limited (ARTC) Engineering Standard Light Signals SPS 11 [7] (now superseded by ARTC ESA-04-01 Colourlight signals and indicators [8]), where results were similar. The authors did not use the conventional names of signalling practices as the set responses were a forced-choice of ‘red’ or ‘orange-yellow’. This is not reflective of signalling practices and does not reflect decisions that rail drivers need to make. They did, however, consider their observations to be relevant to the occurrence of SPADs. Their definition of normal colour vision relied on Ishihara’s test only, and the examination lighting conditions were either not specified [3] or only the illuminance was specified, but insufficient detail was provided on the colour (‘LED office lighting’) [4]. The pass/fail criterion was not stated. It has long been known that the performance on Ishihara’s test is related to the colour of the illumination [9].

Holmes [10] observed that ‘*For example, an observer presented with a bluish-green light and asked to call it ‘green’ or ‘not green’ would almost certainly call it ‘not green’ but if asked to call it ‘green’ or ‘red’, he would call it ‘green’. The limits of the area on the chromaticity diagram called ‘green’ when the choice is ‘green’ or ‘not green’ would be different from those when the choice is ‘green’ or ‘red’. Further, the choice made by the observer will differ according to whether he knows the colour of the light to be one of two or three alternatives, or to whether he is told that the colour may be anything, because in the first problem his decision may be reached by rejecting the alternatives whereas the second problem requires positive recognition of the colour*’.

As a consequence, the current research has not reflected modern Australian signalling practices (which are mostly light-emitting diode (LED)) or the prevailing colour naming (red, yellow and green) into its methodology, so the practical significance of the observations is unclear.

Topley [11] and Cole and Vingrys [12] report visual acuity effects on the performance of the Board of Trade (BoT) Lantern and Holmes-Wright Type B (HWB) lantern, respectively, but the types of misnamings made are not reported. Only one of their 100 subjects had a visual acuity as poor as 6/9, the remaining having 6/7.5 (LogMAR 0.10) or better. Cole and Vingrys report that the BoT Lantern is a stringent test like the HWB lantern. Since it was designed for maritime use, the HWB uses a colour code of red, green and white and required sighting distances are mainly 6 nautical miles (11.1 km) for white lights and 3 nautical miles (5.6 km) for coloured lights [13]. Railways use red, yellow and green for fixed signals, and the critical distance is 1.6 km when locomotive driving [14]. The railways do use red, green and white for their hand-held lanterns. Cole and Vingrys [15] reported that the HWB lantern is more stringent than the Farnsworth Lantern (FALANT), which was also originally developed for naval use and also has a red, green and white code [16]. The FALANT and Railway LED Lantern Test (RLLT) are essentially equivalent in difficulty [14]. In addition, in the previous studies [3, 4], the subjects were presented with single signal lights, whereas suburban signalling practices mostly use double lights. It has long been the practice to design lanterns with two lights [17] or even three [18]. Performance is affected by the number of lights presented [19].

The current study aims to investigate the effects of both refractive and non-refractive defocus on the detection AND recognition of LED rail signals. This current study uses colours and colour names (red, yellow and green) as used in trackside signalling and uses the official colour vision test of the Australian railways.

The RLLT [14, 20, 21] is a simulated practical test nominated in the Australian National Railway Medical Standards (current version [22]) carried out at a test distance of 6 m for those needing to meet normal colour vision standards such as locomotive drivers and at 3 m for those needing to detect and recognise coloured signal lights at shorter distances (e.g. station assistants and trackside workers). It is consistent with modern railway signalling practices, including the use of LEDs that are used in signal construction and a range of luminous intensities representative of railway signals in practice. It is possible that the RLLT could be used to identify those at risk of misnaming red under conditions of defocus. Using the RLLT should be a more valid approach in both a clinical and practical domain when investigating red misnaming, as the colours and luminous intensities in the workplace are replicated in the signal. Use of the RLLT involves the prevailing signalling practices, and accepted responses of the RLLT involve only red, yellow, green and absence of a signal, both as stimuli and responses.

Bangerter filters (Reyser Optik AG, ryseroptik.ch) are translucent stippled plastic filters used to degrade image quality in the treatment of amblyopia. Bangerter filters act more like a Gaussian filter, with which there is monotonically increasing contrast reduction of higher spatial frequencies [23]. The effects of Bangerter filters on colour vision have also not been investigated previously, but may be useful to assess colour perception when contrast is reduced without the effect of chromatic aberration, such as occurs in the presence of ocular pathology. To ensure that the Bangerter filters did not have spectrally specific effects, the procedure reported in the Appendix was carried out. The spectral transmittance (direct and diffuse) of the Bangerter filters used was measured, and the performance was assessed using the criteria of the standards relating to colouration limits for clinical observation [24–26]. These are much more stringent requirements than the colouration requirements for traffic signals in eye protection (e.g. [27]). As an illustration of the insignificance of this spectral transmittance uniformity, blue-blocking lenses, which have a slightly visible tint, have been shown to have no statistically significant effect on colour discrimination [28] and also to comply with these clinical observation colouration requirements [24]. As a consequence, any colour contingent effects of the Bangerter filters are not an inherent property of the filters.

## Methods

### Subjects

Participants were recruited via email to the School of Optometry and Vision Science, University of New South Wales (UNSW), Sydney, by the programme coordinator and utilising notice boards around the university facilities. The study and recruitment process were conducted with ethics approval from the UNSW Human Research and Ethics Advisory Board. The ethics approval number was HC220196.

The inclusion criteria to be included in this study comprise: age 19–59, best corrected visual acuity was no worse than 6/9 (LogMAR 0.17) binocularly (being the requirement in the Australian railway medical requirements), healthy eyes with no current or active ocular conditions and sufficiency in English. The exclusion criteria were an age range outside of 19–59 (being within the working age range for locomotive drivers) and a colour vision deficiency (being a requirement for locomotive drivers). Experienced railway employees were not included in the criteria, as a previous study has shown that naive subjects and railway workers performed equally well on the RLLT [14, 20, 21]. Additionally, at the time of recruitment to this study, it was advised that recruitment of railway employees was not an option due to the industrial action at the time on NSW Railways (Casolin, A. personal communication).

Participants were made aware of the procedures involved in the study and inclusion and exclusion criteria before signing a participant information and consent form prior to undertaking both screening and the study.

### Screening

A screening process was required to determine eligibility to participate in the study. This included normal colour vision status, subjective refraction, best corrected visual acuity, and ocular health assessment. To screen participants for a colour vision deficiency, participants needed to pass ≤3 errors on the screening plates of a 1996 edition 24-plate Ishihara colour vision test administered under a Phillips fluorescent tube source type 965 (Phillips, lighting.philips.com). This source has a CIE general colour rendering index ≥90 and a nominal correlated colour temperature of 6500 K, consistent with recommendations [29, 30]. They were further required to have an anomalquotient in the range 0.8–1.2 and a matching range ≤5 scale units on the Neitz Model OT anomaloscope (Neitz Instruments Co Ltd., neitz.co.jp). This anomaloscope complied with the recommendations for Rayleigh equation anomaloscopes [29, 31, 32]. A full subjective refraction was performed to obtain the patient’s refractive error and best corrected visual acuity. Ocular health assessment was assessed using fundus photography with the iCare DRPlus (iCare Linland Oy, icare-world.com) to examine the posterior pole.

### Procedure

The RLLT (ART Electronics, railway-technology.com) was administered at 6 m, the specified test distance for locomotive drivers [22]. At 6 m, the task represents that of a high-speed country train driver who must detect and recognise a signal at, at least the necessary stopping distance of 1600 m [14]. The test presents 24 pairs of signals that are vertically aligned and each shown, automatically, for 2.1 ± 0.1 s. The colours and luminous intensities are representative of NSW railway practices. Accepted responses for each signal were ‘red’, ‘yellow’, ‘green’ or ‘no signal’. The order in which the signal pairs were presented was randomised. In the clinical application of the test, the signal pairs are at a fixed location and normally administered from 1 to 24 or 24 to 1. The order of the unblurred and blurred presentation sets was randomised. The lantern used was recalibrated annually in an ISO/IEC 17025 [33] accredited laboratory, as required at the time by Transport for NSW.

**Fig. 1:**
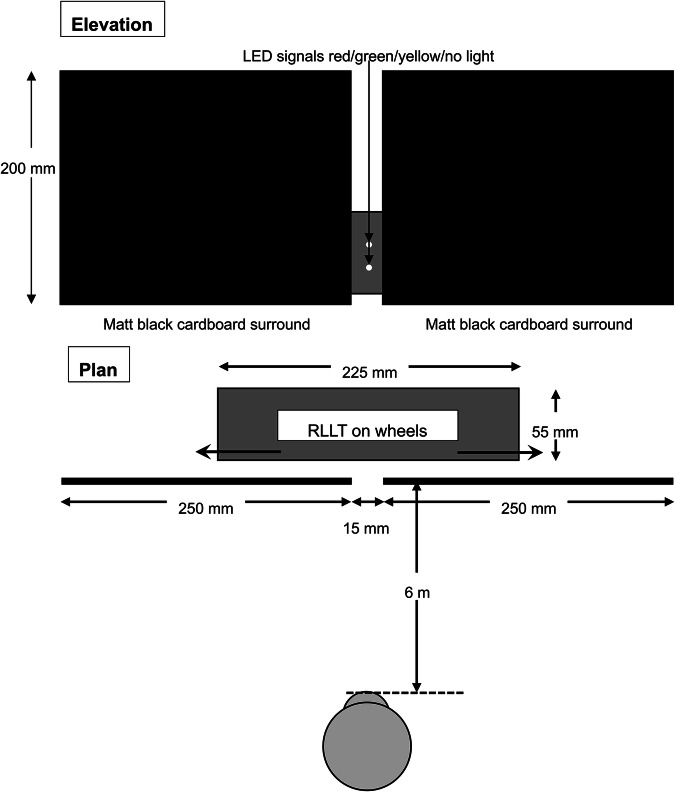
Schematic of the RLLT set-up with occluders; top view (top) and front view (bottom). The lantern is positioned behind a two-part surround so that one pair of lights in the lantern can be displayed without providing a clue to the position within the sequence of lights. The observer was 6 m away, which is the test distance for train drivers. LED light-emitting diode, RLLT Railway LED Lantern Test.

In order to mask the presentation order and prevent memorisation, the RLLT was mounted on wheels to allow lateral movement and placed behind a black cardboard aperture as shown in Fig. 1. This allowed only a single presentation pair to be seen at a constant location. The rest of the instrument was obscured so that the position of the signal pairs in the array could not be determined.

The luminance of the black surrounds, as illustrated in the second part of Fig. 1, was <1 cd m^−2^.

### Test Conditions

The RLLT was administered under five viewing conditions binocularly using trial lenses in a trial frame adjusted to the subject’s distance inter-pupillary distance and set vertically so that they viewed through the centre of the lenses to avoid prism by decentration and lateral chromatic aberration effects. The five viewing conditions were:

1. Best corrected refraction
2. add +0.50 DS binocularly
3. add +0.75 DS binocularly
4. add Bangerter filter 1.0
5. add Bangerter filter 0.8

Conditions 2 and 3 involved an adjustment of +0.50 DS and +0.75 DS in the trial frame (i.e. the power of the trial frame lenses was changed such that there was only one spherical and one cylindrical lens), to avoid the interreflections and transmission losses that extra surfaces would cause. The Bangerter filters for conditions 4 and 5 were adhered to flat glass goggle lenses that were worn over the top of the trial frame. The best corrected refraction was used as a control. +0.50 DS and +0.75 DS were chosen to include the viewing conditions of Wood et al. [3, 4]. Bangerter filters 1.0 and 0.8 were chosen after a pilot study showed that the 0.6 filter and lower were too visually degrading and resulted in an inability to do the test at all. The higher levels of refractive blur used in the previous study [4] were also omitted to reduce the demand on the subjects and because they would reduce visual acuity well below that required in the medical standards [22].

### Procedure

All procedures were performed at the Colour Vision Clinic of the University of New South Wales. The prevailing COVID-19 hygiene protocols were followed.

Visual acuities were measured without and with Bangerter filters 1.0 and 0.8. Contrast sensitivity was measured binocularly using the MARS test (Mars Perceptrix Corporation, marsperceptrix.com) with best corrected refraction followed by added Bangerter filters.

The RLLT was administered according to the instruction manual, with the participant seated 6 m from the instrument in a normally lit room (300 lux) without significant glare and no visible windows. Ambient lighting has been shown to have no effect on the results of the Farnsworth lantern [34].

They were instructed: ‘You will be shown a pair of lights, one above the other, for 2 s. I want you to tell me what colours you see. Tell me the top one first. The only colours you will be shown are red, yellow and green, and these are the only colour names that you can use. In some cases, there is only one light, in which case respond, ‘no light’, again in the order of top first, then bottom’.

Breaks were given after each test to minimise patient fatigue.

‘Red-signal-related errors’ were defined as incorrect responses involving the signal red. These included any missed red, red reported as yellow and red reported as green. The outcome for red-signal-related errors was the proportion of the total number of red presentations.

## Results

A total of 20 participants completed the study. Table 1 provides participant demographics. Table 2 provides details of visual acuity in the five states. The subjects all had better than LogMAR 0.20 (≈6/9) visual acuity, the worst being one case of LogMAR 0.02 (6/6^−1^).

**Table 1:** Demographics of the study participants (mean ± 1 standard deviation).

Characteristic	Value (mean ± 1 s)
Age (years)	22.4 ± 1.1
Gender	
Male, *n* (%)	10 (50)
Female, *n* (%)	10 (50)

**Table 2:** LogMAR visual acuity of the participants.

	Best corrected	Bangerter		Refractive blur	
		1	0.8	0.50 D	0.75 D
Mean	−0.11	0.05	0.23	0.17	0.30
SD	0.06	0.09	0.14	0.08	0.10
Median	−0.10	0.05	0.23	0.18	0.31
1st quartile	−0.12	−0.02	0.14	0.09	0.21
3rd quartile	−0.08	0.10	0.30	0.23	0.38
Skewness	−0.11	0.37	0.40	0.35	0.42

Absolute values of skewness ≤1.0 indicate a normal distribution [59].

*SD* standard deviation.

**Fig. 2:**
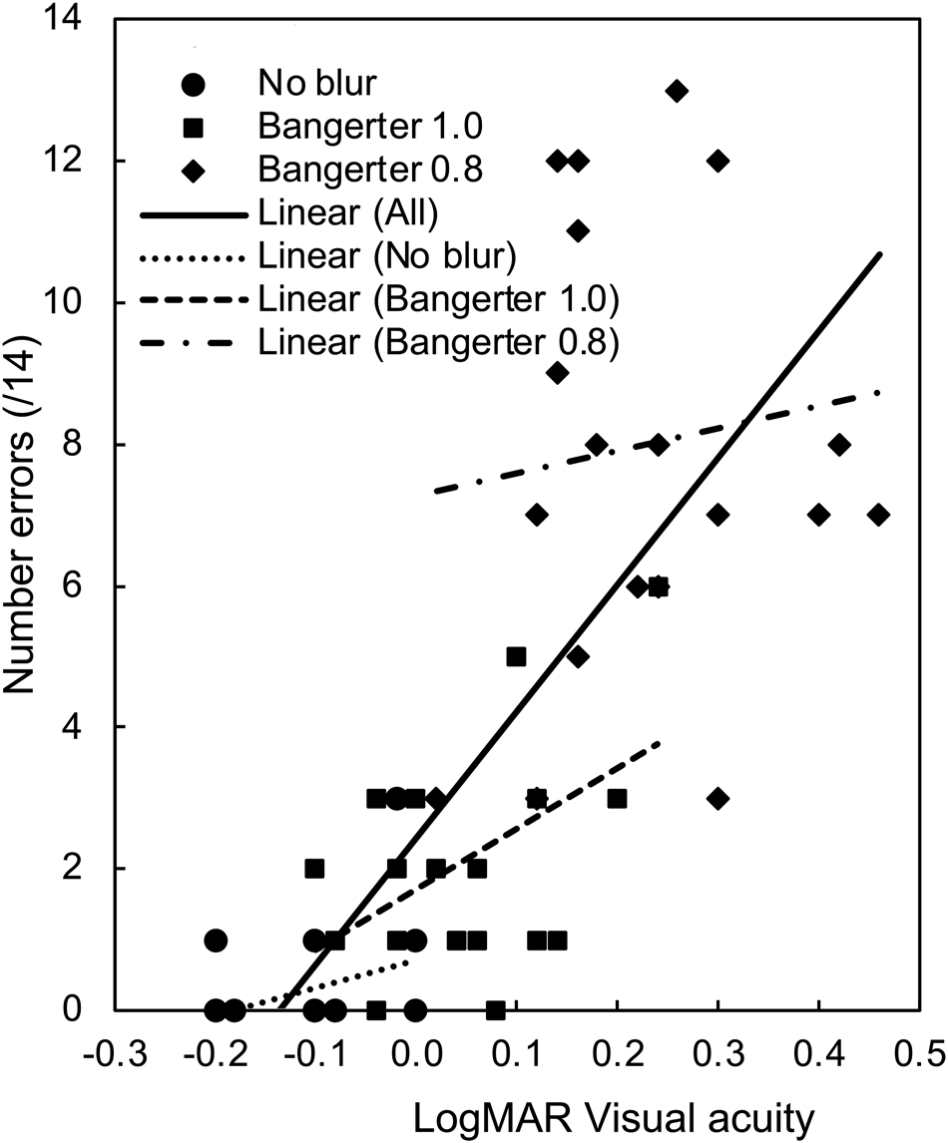
The relationship of LogMAR visual acuity and number of red-naming errors (out of 14). No blur, Bangerter 1.0 and 0.8 results are shown as circles, square and diamond symbols, respectively. The linear regression lines are shown as dotted, dashed and dot-dash lines, respectively, and the linear regression line for the combined values is a solid line.

Figure 2 shows the relationship between the number of errors made naming red, LogMAR visual acuity and Bangerter-induced blur, as an example. Table 3 lists the Pearson correlation coefficients for each level of blur and the significance of the value.

**Table 3:** Correlation coefficients for the relationship between visual acuity and number of red-naming errors for each blur type and for the results as a whole.

	Red errors			
	No blur	B 1.0	B 0.8	All
Correlation	0.327	0.420	0.107	0.745
Significance	*p* = 0.17	*p* = 0.07	*p* = 0.64	*p* < 0.01

**Fig. 3:**
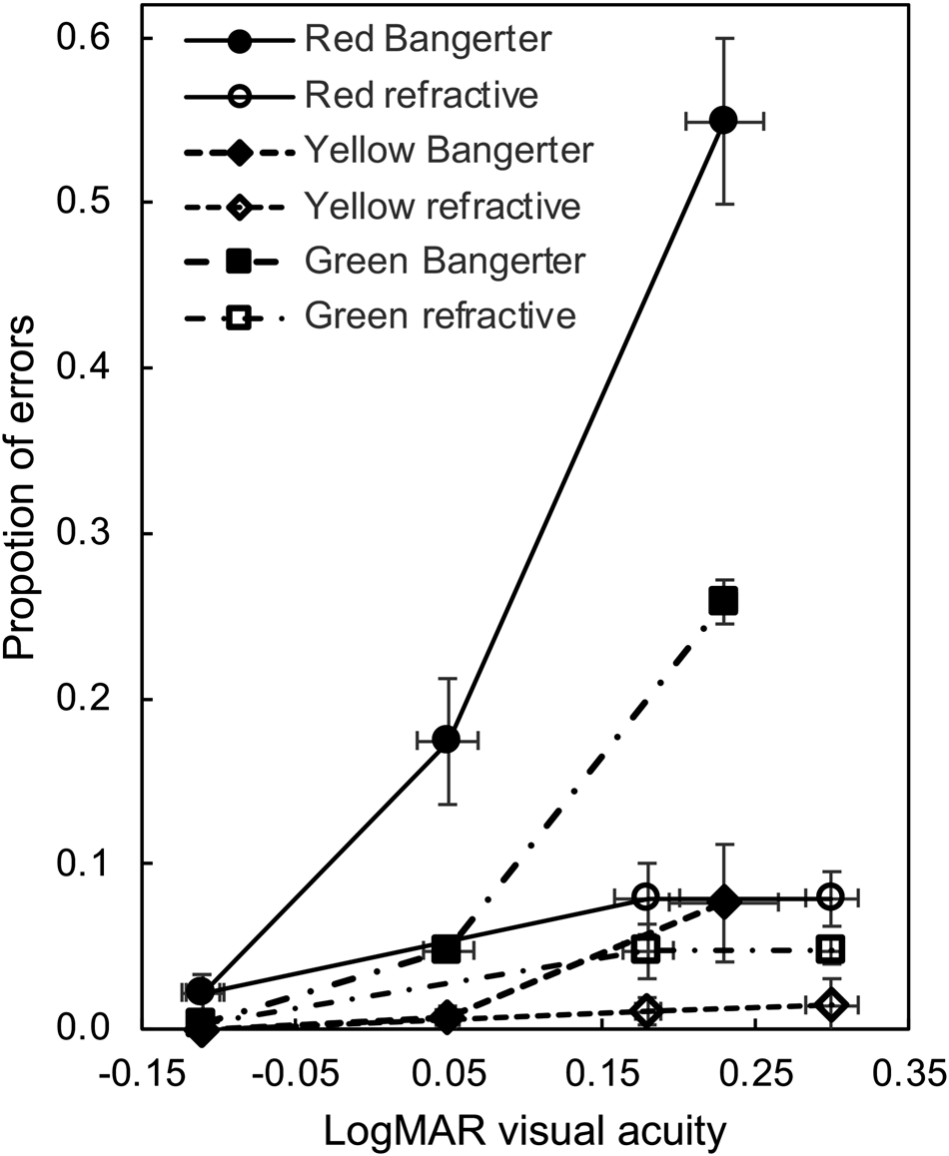
Relationship between mean LogMAR visual acuity and mean colour naming errors for each signal colour and non-refractive (Bangerter filter) and refractive blur. Error bars are ±1 standard error in proportion of errors and visual acuity. The *Y* error bars may be smaller than the symbol.

Figure 3 shows the proportion of errors made on each of the signal colours; these comprise all the errors for each signal colour, that is, signal misnamed or not seen. The responses where the subject reported a colour when none were present are not included. This error occurred only once in the unblurred state; 3 subjects each made 1 error in each of the refractive blur states, there were no errors in the lesser Bangerter 1.0 blur state, and 2 subjects made an error and 1 subject made 3 errors in the Bangerter 0.8 non-refractive blur state.

The same analysis has been carried out on the yellow and green errors. There were no significant relationships found between the number of errors made and visual acuity within the three induced blur levels of each blur type, but there was a significant relationship, *p* < 0.01, for the pooled data between errors made and visual acuity. For the pooled data, the results are set out in Table 4.

**Table 4:** Correlation coefficients and significance for the relationship between the number of naming errors and visual acuity for each blur type.

	Errors per subject		
Blur type	Red	Yellow	Green
Bangerter	0.745	−0.074	0.546
	*p* < 0.01	*p* = 0.94	*p* < 0.01
Refractive	0.288	−0.088	0.375
	*P* = 0.02	*p* = 0.943	*p* < 0.01

**Fig. 4:**
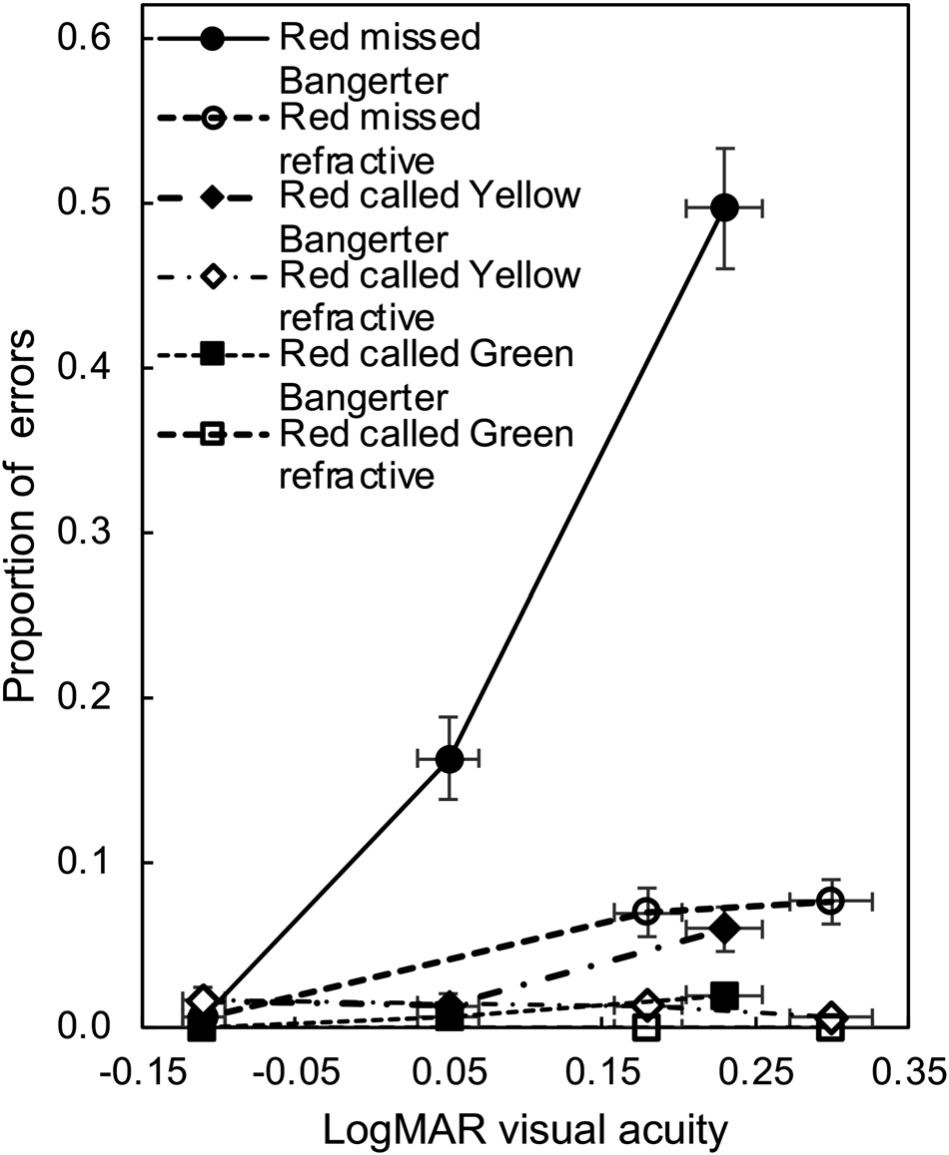
Relationship between mean LogMAR visual acuity and mean red signal errors for non-refractive (Bangerter filter) and refractive blur. Error bars are ±1 standard error in proportion of errors and visual acuity. The *Y* error bars may be smaller than the symbol.

Figure 4 shows the breakdown of red-signal-related errors, that is, when a red signal is missed or red is miscalled as yellow or green.

Errors occurred far more often with red than with yellow or green (*p* < 0.0001) and with Bangerter filter blur more than refractive blur (*p* < 0.0001). Failing to see a red signal, rather than misnaming the red as yellow or green, was the most predominant error (*p* < 0.0001) and was far more easily induced by the Bangerter filter than refractive blur (*p* < 0.0001) and is a lot less common than miscalling red as yellow (*p* < 0.0001) (paired *t*-tests).

## Discussion

The predominant effect of both types of blur is to cause the observers to miss the red signals rather than misname them. While miscalling red as yellow is the second most common error, it is very much less frequent than failing to see the red. The not-seen rate in the previous study was not reported [3]. The frequency of red misnamed as orange-yellow was rare (about 9.4% for the 1030 cd m^−2^ background and 0 misnamings for the 382 and 100 cd m^−2^ backgrounds) for the +0.75 D blur condition and dimmer backgrounds, which was similar to the present study results for the same blur level. The previous studies [3, 4] used both bright and dim backgrounds, with different misnaming rates. In addition, the second clear effect is that the Bangerter filters, despite causing less loss in visual acuity than refractive blur, give rise to more colour naming errors of all types. This may be seen by comparing the filled and unfilled symbols in Fig. 3. Bangerter filters also give rise to more scattered light than ophthalmic lenses (see Appendix), so that the less immediate background of the room lit to 250 lux maybe more effective in masking the signals.

The results show that responses with refractive blur show fewer errors than the bright surrounds of the previous studies, where a significant effect of refractive blur on the miscalling of red as ‘orange-yellow’ was reported. While the authors wrote about red looking yellow, the actual experiments did not permit the use of ‘yellow’ alone as a descriptor. From the earliest studies of colour naming, much variation has been shown in experimental results. McNicholas [35] used both red/orange/yellow/white/green/blue/purple and red/orange-yellow/white/green/blue colour name sets. The diameter of his stimuli was varied to provide equal luminous intensity and was variable in the range 0.04–0.36 min of arc. At about 608 nm, the stimulus was called ‘red’ and ‘yellow’ in equal proportions (each about 14% of responses), but the predominant name used (about 72%) was ‘orange’. When ‘orange’ and ‘yellow’ responses were replaced by ‘orange-yellow’, the overlap of ‘red’ with ‘orange-yellow’ was substantial, and these names were used equally frequently at about 618 nm. His data on ‘green’ are confounded by the inclusion of ‘white’ as a response. There was no test to establish that the observers had normal colour vision. Hill [36] used fields of 0.5 min of arc and Ishihara’s test as proof of ‘normal’ colour vision. When his subjects showed a ‘psychological reluctance’ to distinguish ‘yellow’ and ‘orange’, he simply combined those two responses. Beare [37] showed that, in 2° fields and given the choice of colour names of ‘red’, ‘orange’ and ‘yellow’, the use of the term ‘red’ falls to 0% at 605 nm and ‘yellow’ falls to 0% at 602 nm, but ‘orange’ overlaps significantly with both. The introduction of the option of ‘orange’ creates considerable overlap, such that around 620 nm is called ‘red’ and ‘orange’ approximately equally frequently and around 570 nm is called ‘yellow’ and ‘orange’ equally frequently. The distinction is less clear for ‘low intensity’ stimuli. The basis for ‘normal’ colour vision included anomaloscopy [38].

Holmes [10, 39] used stimuli between 0.7 and 7 min of arc and collected data on ‘orange’ but did not report any. He reports that ‘Experiments in the differentiation between yellow and orange showed considerable confusion between these colour names at low illuminations’. He gives no method for the establishment of ‘normal’ colour vision in his subjects. Soon and Cole [40], using stimuli of 1 and 7 min of arc, did not use the response ‘orange’ either. In addition, their Red 2 and Red 3, see their Table 2, are too orange (*y* > 0.292) to comply with the prevailing requirements [8] for wayside signals, although they do comply with the requirements for highway/rail crossing (being the same as for traffic signals generally [41, 42]). Red 1 was rarely called ‘yellow’ by young (aged 18–28 years, mean 23.0 years) or old (aged 50–64 years, mean 54.6 years) subjects. Similarly, Yellow 2 and Yellow 3 are too green to comply (*y* > 0.430). So there is a blurred borderline between red and orange, and between yellow and orange that does not exist between yellow and red. They also used Ishihara’s test to define ‘normal colour vision’.

In the first Wood et al. study [3], the subjects had the option to report a signal as not seen. Whether or not the error occurred was not explicitly reported. This option was not provided in the second study [4]. The problem here is that if a signal is not seen, then the subject will be required to guess and will choose a response at random. So, half the time, a failure to see a signal will result in a two-alternative forced-choice response of the wrong colour. Thus, miscalling of red as ‘orange-yellow’ would have occurred 50% of the time that the signal was not seen. Since the yellow has about twice the luminous intensity of the red in their experiments, not seeing the yellow will occur significantly less often. In the current study, participants were given options between ‘red’, ‘yellow’, ‘green’ or ‘no light’. This is reflective of signalling practices and represents the task that train drivers are given, and reds that are not seen will not be called ‘yellow-orange’.

**Fig. 5:**
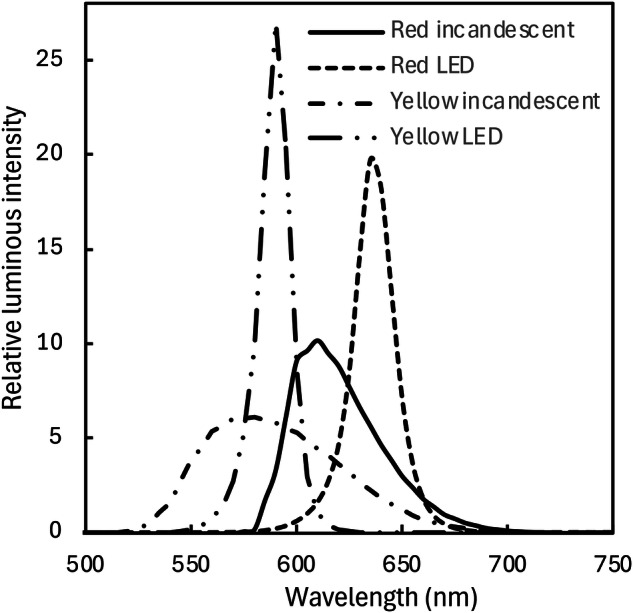
Relative spectral luminous energy of incandescent and LED traffic signals. The values have been equalised for total luminous energy. Plotted from data in ISO 18526-2:2020, Annex D [43]. LED light-emitting diode.

If the effects are related to chromatic aberration, the narrower band spectral distributions of LEDs will give rise to less visible effects. The narrower spectral bandwidth of LEDs compared with incandescent signals with a broad band filter is well illustrated in Fig. 1 of the earlier previous study, as is the narrower spectral distribution of the interference filter [3]. The authors did not separate the responses for the broad band and narrow band red for the 5 m viewing distance, but did state that the narrow band stimulus was too dim to see at the 10 m viewing distance with positive blur. The 10 m viewing distance was scaled to represent a 1 km viewing distance, which was closer to the RLLT scaled value of 1.6 km. The subjects’ inability to see the red signal was consistent with our results under viewing conditions near the signal’s threshold. They also commented that the hue shifts occurred for both the LED and incandescent signal lights in their field observations, indicating that there is nothing unique about the spectral distribution of LED signal lights. The differences in incandescent and LED sources may also be seen in the traffic signals used in the ISO test method standard for eye and face protection [43], see Fig. 5. Thus, any chromatic aberration effects [44, 45] (longitudinal or lateral) may be different for the two types of signal.

The phenomenon was originally described [3] as occurring when signals were blurred because they were viewed through the intermediate corridor of a progressive lens, although it was noted that blur in general also gave rise to the observation. The authors discounted ‘chromatic aberration’ as an explanation because it was in the wrong direction to explain the results, since red is focussed, in the refractively blurred condition, closer to the retina, not further away. This argument assumes focus is accurate without any leads of accommodation, as would occur, for example, in darker environments, in a phenomenon known as empty field myopia [46]. Wood et al.’s argument implicitly addressed longitudinal chromatic aberration, but they were not specific [3].

As an anecdotal comment, some years ago, a request for resolution of a complaint came to the Optics & Radiometry Laboratory, which is the only testing laboratory in Australia that is ISO/IEC 17025 [33] accredited to test to the eye and face protection standards. The complaint related to two prescription eye protectors that were supposed to be identical, but the newer pair caused the wearer to see coloured fringes, and the older pair did not. Both pairs were the same material (polycarbonate), the same design of progressive lens, the same distance prescription and near addition, the same frame, the same base curves and the same centration distances. What was identified as the only difference was that the newer pair had prism thinning applied in manufacture. This is a process in which equal vertical prisms are worked on the two lenses to reduce the thickness and make the lenses lighter, and minimise edge thickness around the superior edge. Polycarbonate is widely used in eye protection, being the most impact-resistant of the lens materials [47]. Complaints about coloured fringes, especially associated with higher refractive powers, are, anecdotally, relatively common with polycarbonate, given that it has the lowest Abbe number of all the ophthalmic lenses (30 compared with 58 for hard resin, allyl glycol carbonate). As a consequence, the phenomenon originally described by Wood et al. [3] may be related to lateral chromatic aberration from the prism, and the blur effect may be coincidental.

**Fig. 6:**
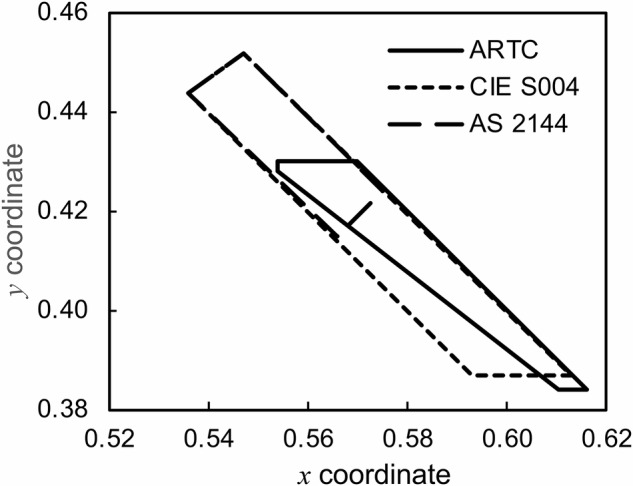
Chromaticity diagram according to the 1931 CIE coordinate system showing for yellow LED lights. The limits set by the Commission Internationale de l'Eclairage (CIE) [48], Australian Rail Track Corporation (ARTC) [7] and the Australian traffic signal standard [41] are indicated. ARTC Australian Rail Track Corporation Limited, LED light-emitting diode.

The numerical specification of the colours and luminous intensities of the RLLT stimuli is considered commercial in confidence, but the colours were chosen to comply with CIE S 004 [48], see Fig. 6. The CIE limits are a little more liberal in the green direction than the ARTC limits [7], but the colours used in the previous study [4] would comply. However, also shown are the yellow limits for traffic signals in Australia [41]. These may be seen to be much more restrictive in the red direction compared with both the CIE and ARTC. So the subject group will comprise people who are familiar with the less orange road traffic signals, which may influence their use of the term ‘orange-yellow’.

The luminous intensities of the stimuli in the RLLT were deliberately varied to reflect the real-life situations where the viewing distances may vary and the observer may not be in the direction of the maximum intensity. While the actual specifications of the signals are considered commercial in confidence, they were derived after making measurements on all the models of signal made available by Railcorp NSW (now incorporated into Transport for NSW transport.nsw.gov.au), at the time. It is fair to say that the luminous intensities will, on average, be significantly lower than the stimuli in the Wood et al. study [4] and closer to threshold. This may be especially so of the red stimuli. Assessment of the original prototype form of the RLLT showed that sometimes normals missed two of the red stimuli, and the intensities were increased so that a second experiment showed that these errors by colour vision normals were no longer seen [14]. It may be that the Bangerter filters reduce the peak intensity more than refractive blur, given the same visual acuity.

The use of Bangerter filters was implemented in this study to investigate whether longitudinal chromatic aberrations are involved in the colour misperception effect. Bangerter filters do not refract light but behave more like a Gaussian filter; thus, longitudinal chromatic aberrations cannot be induced. A study by Gupta et al. [44] demonstrated that longitudinal chromatic aberrations and not monochromatic aberrations may be linked with the colour misperception phenomenon. This effect is also not affected by the L/M cone ratio. They also proposed that an additional defocus-dependent neural mechanism may also contribute to the change in colour appearance [48]. Hence, the presence of missed reds and reds called yellow is indicative that factors other than longitudinal chromatic aberration may be inducing a colour misperception effect. The presence of red misperceptions with the Bangerter filters in this study raises the question as to whether a non-optical defocus mechanism is involved, and this merits further investigation.

The errors made by participants under best corrected conditions are anomalous in that 2 participants (10%) failed the RLLT. In the development study [13], 106 colour vision ‘normals’; only one (0.9%) individual failed. At the time, a pass on the RLLT constituted less ≤ two misnamings, ≤ one green or yellow missed and ≤ one blank named as a colour. In the commercially available version and based on a decision by RailCorp NSW (now incorporated into Transport for NSW), any missed or miscalled reds constitute a fail. However, in an experimental domain, naive participants may not invest sufficiently to avoid making judgement errors. This is known as the mutable-zero effect in risky choices [49].

There are probably many factors leading to the occurrence of a SPAD. These could be due to a signal being missed, being misperceived or some other factor. There does not seem to have been any consideration of this issue in any of the studies of causes of SPADs. In the case of the NSW railways, the investigation of SPADs does not include direct questions about whether the signal was not seen or seen as yellow (Casolin, A. personal communication), and there are no reports from other jurisdictions to be found. As a consequence, the hypothesised [4] association between red signal errors (missing or misnaming) and SPADs must be seen as very tentative at this stage.

At the time of the first Wood et al. study [3], the manufacturing standard on prescription eye protection, AS/NZS 1337.6 [50], was yet to be published, and the conventional wisdom was that polycarbonate was the material of choice for medium impact occupational eye protection. ‘Medium impact’ in AS/NZS 1337 was the same, at the time of Wood et al. [3], as ‘low energy impact’ in EN 166:2001 [51] and ‘high velocity impact’ for spectacle-type eye protectors in ANSI Z87.1:2003 [52]. Since that time, other lens materials for this level of impact protection have come into use. While polycarbonate remains the most impact-resistant material [53], there are other materials (most notably polyurethane, introduced in 2001) that do comply with the medium impact requirements, have a lower refractive index and a higher Abbe number than polycarbonate. So, if the coloured fringing is an issue, there is, these days, an alternative solution.

## Conclusions

Rather than a colour misperception, the errors seem to be more related simply to missing the signal when there is minimal stray light from the background. This current study does find that low refractive blur and non-refractive blur causes red, more than yellow or green, to be missed rather than misnamed. This study also shows that non-refractive blur is a more potent means of inducing error in colour detection and, to a lesser extent, recognition in a simulated practical test.

There are several methodological reasons why this study gives different results from the earlier studies [3], given the difference in the stimuli and, especially, in the responses required of the experimental subjects. It was suggested that stray light and the Abney effect could [3, 4] play a role in the misnaming since the desaturation of red leads to an increased likelihood of naming as yellow. Abney observed that desaturating (adding white) to a spectral colour could change the perceived hue. In particular, red was more likely to be called orange even though the dominant wavelength had not changed. Thus, the bright surround was associated with the errors that did not occur in this study and the earlier studies when the light was presented within a dimmer background. Adaptation is also affected and affects colour naming in a complex way [54]. The fact that the red signals may be closer to threshold than the yellow or green may also be a factor.

Given that the RLLT is a simulated practical test, the results of this study suggest that the conclusions of the original observations [3, 4] of miscalling red as ‘yellow’, having practical consequences, may be overstated. Until the post-SPAD de-briefing questions include the differentiation of ‘red signal not seen’ and ‘red signal not perceived as red’, the link is unlikely to be resolved. Even though the Goodwell, Oklahoma, collision [55] involved a SPAD and the vision and colour vision of one of the engineers were identified as inadequate, the incident involved failing to reduce speed to 40 mph in response to a flashing yellow, failing to reduce speed further to 30 mph in response the yellow over red signals and, finally, failing to stop, or even slow down, at a red signal. Even misperceiving the red as yellow should have led to a slowing of the train. So this collision was preceded by failing to see red and yellow signals, with the number of lights being redundant information that should have resulted in a response even if the colour was misperceived.

Since the Australian medical standards [22] also include a requirement for visual acuity (6/9 LogMAR 0.17 in the better eye), this may be an indirect way of minimising this issue in practice.

The extent to which the results from a young population may be extrapolated to older observers is not known, but Soon and Cole [40] showed no age effect in the recognition of the only red, in their set of 3 reds, that was rail-use compliant.

## Data Availability

The data that support the findings of this study are available from the corresponding author, [SJD], upon reasonable request.

## Appendix: Evaluation of Spectral Transmittance of Bangerter 1.0 and 0.8 Filters

To evaluate the possibility that the spectral transmittance of the Bangerter filters might be significantly non-uniform and might influence the results of a colour naming experiment, the following examination methods were applied.

### Methods

These measurements were carried out in ORLAB in the School of Optometry and Vision Science, University of New South Wales. ORLAB is ISO/IEC 17025 [33] accredited by the National Association of Testing Authorities for these measurements.

The spectral transmittances of the two Bangerter filters were measured and analysed according to the method that is common to ISO 12609-1 [26] and AS 16321.4 [56] and validated as providing no significant interference with clinical observation of the colour of patients [24]. This is the most stringent requirement used in eye and face protection standards and is substantially more stringent than the colour signal detection criterion found in sunglass, eye and face protection and ophthalmic lens standards.

The total and diffuse spectral transmittances were measured using an Agilent (www.agilent.com) Varian Cary 5000 UV-Vis-NIR dual beam, double monochromator spectrophotometer (ORLAB ID #278) fitted with a diffuse reflectance attachment (ORLAB ID #137). The wavelength accuracy has been established with spectral lines as ±0.1 nm. The uncertainty of measurement (UNC) of spectral transmittance has been established with filters (ORLAB #243) for which the spectral transmittances have been provided by the National Research Council of Canada (nrc.canada.ca) (NRC Calibration Report PAR-2026-3363 dated 25 October 2026).

$${\mathrm{UNC}}(\lambda ) \% =0.1+0.002\cdot \tau \left(\lambda \right)+0.1\cdot \frac{{{\rm{d}}}\tau }{{{\rm{d}}}\lambda }$$

Where *τ*(*λ*) is the % spectral transmittance at wavelength *λ*.

Measurements were made at 1 nm intervals with a spectral half-power bandwidth of 1 nm.

The mean and minimum spectral transmittance (450–650 nm) and the minimum to mean ratio were calculated.

The haze of each filter was measured using a Drick Instruments DRK-WGT-2S haze metre (ORLAB #411). This provides haze and luminous transmittance measurements for CIE Illuminant D65 (ISO 14782 [57]) and CIE Illuminant A (ASTM D1003 [58]). The metre has been calibrated using a set of six haze reference artefacts for which haze values (Illuminants A, C and D65) have been provided by the National Physical Laboratory, UK. (www.npl.co.uk) (NPL Report 2016040307 dated 27 October 2016). The uncertainty of measurement is ±0.2% absolute.

### Results

Results for mean and minimum spectral transmittance and the ratio of the two, with a compliance statement, are set out in Table 5.

**Table 5:** Spectral transmittance characteristics of the Bangerter filters 1.0 and 0.8 and the colouration requirements of ISO 12069 [26] and AS 16321.4 [56].

	Bangerter filter 1.0			Bangerter filter 0.8		
	% spectral transmittance in range 450–650 nm					
Measure	Total	Diffuse	Direct	Total	Diffuse	Direct
Mean	87.6	2.2	85.4	87.9	5.0	82.9
Minimum	86.4	2.0	83.9	86.6	4.8	81.2
Minimum/Mean	0.99	0.94	0.98	0.99	0.97	0.98
Requirement (ISO 12609-1 [26] and AS 16321.4) [56]	≥0.80					
Compliance	Pass	Pass	Pass	Pass	Pass	Pass

Results for the haze of the two Bangerter filters are set out in Table 6.

**Table 6:** Wide-angle scatter (haze) measurements of the Bangerter filters 1.0 and 0.8.

CIE Illuminant ISO 11664-2 [60]	Measure	Bangerter filter 1.0	Bangerter filter 0.8
D65 (phase of daylight)	Haze (wide-angle scatter) %	2.8	9.5
	Luminous transmittance %	90.8	90.9
A (tungsten filament lamp)	Haze (wide-angle scatter %)	2.8	9.4
	Luminous transmittance %	90.9	91.0

### Discussion

The ratios of minimum to mean transmittance (total, diffuse and direct, 450–650 nm) are very close to 1.0 and easily comply with the ISO 12609 and AS 16321.4.

The haze and luminous transmittances to CIE Illuminants D65 (a relatively blue illuminant) and A (a relatively yellow illuminant) are, within the uncertainty of measurement, the same. This is a further illustration of the neutrality of the transmittance of the Bangerter filters.

### Conclusion

The spectral transmittance characteristics of Bangerter filters will not cause any significant effect on the visibility of the red, yellow and green signals.
